# What Women with HIV Know about Heart Health and Cardiovascular Risk and Intervention Preferences

**DOI:** 10.3390/ijerph21091149

**Published:** 2024-08-29

**Authors:** Lunthita M. Duthely, Sanjana Satish, Sapna A. Kedia, Lilliana Vilchez, Priscilla T. Valls, Michaela E. Larson, Carolina Cruzval O’Reilly, Vanessa Hurtado, Maria Camila Bernal, Karla Inestroza, Nicholas F. Nogueira, Tiffany R. Glynn, Mariano J. Kanamori, Claudia A. Martinez

**Affiliations:** 1 Department of Obstetrics, Gynecology & Reproductive Sciences, University of Miami Miller School of Medicine, Miami, FL 33136, USA; 2 Department of Public Health Sciences, University of Miami Miller School of Medicine, Miami, FL 33136, USA; lcv45@miami.edu (L.V.); m.larson4@umiami.edu (M.E.L.); mkanamori@med.miami.edu (M.J.K.); 3 Medical Education, University of Miami Miller School of Medicine, Miami, FL 33136, USA; sanjana.satish@med.miami.edu (S.S.); sxk356@med.miami.edu (S.A.K.); n.fonsecanogueira@umiami.edu (N.F.N.); 4 Division of Cardiovascular Medicine, University of Miami Miller School of Medicine, Miami, FL 33136, USA; ptv7@med.miami.edu (P.T.V.); cmartinez5@med.miami.edu (C.A.M.); 5 School of Medicine, Universidad Central del Caribe, Bayamon 00956, Puerto Rico; 123ccruzval@uccaribe.edu; 6 Department of Cardiovascular Disease, University of South Florida, Tampa, FL 33606, USA; vahurtado@usf.edu; 7 Department of Family Medicine, Baptist Health Medical Group, Miami, FL 33143, USA; maria.bernal@baptisthealth.net; 8 Department of Cardiology, Mayo Clinic, Rochester, MN 55905, USA; inestroza.karlalilibeth@mayo.edu; 9 Department of Emergency Medicine, Brigham and Women’s Hospital, Boston, MA 02115, USA; trglynn@mgh.harvard.edu; 10 Department of Psychiatry, Massachusetts General Hospital, Boston, MA 02114, USA; 11 School of Medicine, Harvard University, Boston, MA 02115, USA

**Keywords:** HIV, women, cardiovascular disease, qualitative

## Abstract

Cardiovascular disease (CVD) is a significant health concern influenced by various determinants. Stigma and resilience have emerged as factors in CVD development and management. Women with HIV (WWH) have higher CVD rates than women without HIV. To improve cardiovascular health for WWH, a comprehensive understanding of how these factors interact, the understanding about individual awareness and willingness to engage in risk-reduction interventions are needed. **Methods:** As part of a study examining CVD risk among WWH aged >35 years old, 90-min focus groups were conducted (May 2022) in the English language. Focus groups aimed to elicit participants’ CVD risk knowledge and potential prevention strategies. Transcripts underwent a qualitative analysis. **Results:** Nineteen WWH participated in three focus groups. Participants experienced the following: (a) enacted stigma related to their HIV diagnosis (e.g., family, church member, healthcare staff); (b) a recent event (e.g., hospitalization of self/family, death in family, chest pain) triggered both heart health-promoting lifestyle changes and suboptimal health behaviors (e.g., COVID-19 pandemic: unhealthy snacking). Participants wanted to obtain more knowledge (“on a mission”) about CVD risk. In total, 100% expressed willingness to take medication or embark on other lifestyle changes to prevent future CVD events. Although participants identified preventative heart health behaviors (e.g., eating healthy foods; exercising; limiting stress, substances, and smoking), misconceptions were also identified (e.g., “catching” heart disease). **Conclusions:** Understanding the interplay of the different factors related to heart health is needed both at the provider and the patient level to inform interventions that reduce CVD risk amongst racial/ethnic minoritized women with HIV, living in the Southern region of the US.

## 1. Introduction

Persons with HIV can expect to live as long as persons not living with HIV, given that they take their antiretroviral treatment (ART) medications regularly and maintain low and stable HIV viral loads. However, the risk of other conditions and diseases increases with age, stemming from inflammation and stress responses that occur at the cellular level [1]. In the US, persons of color (i.e., Black, Indigenous, Hispanic/Latinx) are more likely to experience psychosocial stressors driven by discrimination and stigma—additional factors that also contribute to HIV progression. At the individual level, a person’s resilience, or coping methods may mitigate suboptimal HIV-related health outcomes driven by such marginalization [2,3].

Compared to their cisgender male counterparts, cardiovascular disease (CVD) risk is elevated for cisgender women with HIV (WWH) [4,5]. To note, transgender women are at elevated risk for both HIV and CVD; however, the focus of the current study was cisgender women—transgender women have unique biopsychosocial risk factors that are important to consider for informing CVD interventions [6]. Additionally, it has been found that psychosocial stress correlates to increased CVD among women [7]. Awareness of CVD risk and willingness to engage in risk-reduction interventions are both needed to improve cardiovascular health for WWH, especially racially minoritized WWH [8]. Another important consideration for persons with HIV is the lifetime dependence on ART to maintain optimal HIV health, and multiple medications (polypharmacy) are sometimes needed to manage comorbid conditions [9].

Elevated CVD risk, specifically, has been reported amongst persons with HIV [10], which is more common amongst racial and ethnic minoritized populations. It has been reported that modifiable risk factors—the factors that can be changed at the individual level—play a large role in CVD risk [11]. Therefore, interventions should focus on these modifiable factors. A recent study found that, compared to non-Hispanic White persons, non-Hispanic Black and Hispanic persons had a higher risk of modifiable cardiovascular risk factors [12]. In the same study, it was found that non-Hispanic Black persons and Hispanic persons also had a greater risk of hospital readmissions due to cardiovascular events and heart failure [12].

Within the context of HIV, a recently reported study also conducted in South Florida, reported that a higher proportion of WWH developed lipid disorders, compared to their male counterparts [13]. Specifically, co-morbidities in the form of metabolic disorders [14] have been associated with long-term use of ART; additionally, metabolic disorders have been shown, longitudinally (up to 3 years), to increase CVD risk [15]. Some of these risks are exacerbated amongst women. In a recent study, hypertension and/or DM was more common amongst WWH compared to men (75% vs. 25%); the risk was heightened for women ≥ 5 years on ART (*p* = 0.0011) [16].

The Southern US is the region where the HIV burden is the greatest and stigma is a contributor to suboptimal HIV-related outcomes. Women in the Southern US expressed that stigma related to HIV was experienced in various settings and life contexts, including medical settings. They did not feel supported in different settings of their lives and these stigma experiences impeded disclosure of their HIV status to others [17]. Another important consideration is the multiple, or intersectional, stigma experiences, expressed by women of color living in the US [18] and in Canada [19].

Among a multi-cultural cohort of women in Southeast Florida, we reported on the correlation between higher levels of HIV-related stigma and higher levels of depression, HIV viral non-suppression and lower levels of resilience [20]. Similarly, a larger study conducted in the Southeast United States reported that HIV-related stigma had negative effects on women’s health, and resilience mediated, or buffered, these effects [21].

The purpose of the current study was to explore, qualitatively, knowledge of CVD risk factors, willingness to engage in activities to decrease their CVD risk and, also, participants’ experiences of HIV-related and other stigmas. The ultimate goal was to identify intervention targets to mitigate CVD risk amongst non-Hispanic Black WWH and Hispanic WWH. To our knowledge, this is one of the first qualitative studies to examine stigma within the context of cardiovascular risk and disease.

## 2. Methods

### 2.1. Participants

The participants were cisgender women, 35 years or older, living with HIV, on stable ART for more than 6 months and without a previous diagnosis of CVD and fluent in the English language (n = 19). The majority (80%) self-identified as Black; 16% as Hispanic/Latina; and 4% as other racial and ethnic groups.

### 2.2. Focus Groups

A total of 3 (n = 5; n = 10; n = 4) ninety-minute focus groups were held in Miami, FL, USA, May 2022, in the English language, with a total of N = 19 participants. The interviews were scheduled originally to take place in person; however, due to institutional restrictions placed during the COVID-19 pandemic, the interviews were held remotely. To ensure equitable access for the patients and any privacy concerns they might have, participation options included telephonic access (local call), or logging into a free, commercially available video conferencing system (Zoom for Healthcare) via mobile phone, tablet or computer; participation via video was optional. Most participants opted for the video option of the conferencing system. A few (n = 4) logged into the meetings telephonically. With permission, interviews were recorded.

The focus groups were facilitated by three members of the research team. One primary- and one co-facilitator was designated for each group. The technical assistant, who was also the assigned note taker, was also available to serve as a backup co-facilitator, should one of the two facilitators encounter technical difficulties. To maximize the comfort of participants taking part in the group and to maximize participation, each focus group began with an “ice-breaker” activity. Participants were provided financial incentives (USD 25) to compensate them for their time. The incentives were available for pickup, beginning the day of the interviews.

Focus groups elicited current knowledge and opinions about CVD risk and potential interventions, with the ultimate goal of identifying barriers to implementation. Approximately 10 pre-generated questions were used to prompt for information. Participants were probed about their interest to learn about their cardiovascular health, their preferred types of prevention and intervention, and optimal dissemination methods for future interventions.

### 2.3. Analysis

The video conferencing system generated the original text from the focus group recordings, which were then individually transferred to the qualitative data analysis software (Dedoose). Machine-generated data instances were reviewed independently by two coders. Instances were identified, consolidated and promoted to key themes. Codes were reviewed further by two other coders. Generally, we used an inductive approach to the coding, which we applied to pre-defined questions that were posed during the focus groups.

## 3. Results

### 3.1. Heart Health Knowledge (See Table 1)

The focus groups began with questions regarding participants’ knowledge of their heart health. Participants shared several preventative measures they knew of to preserve or improve their heart health. These included the use of specific food or vitamins, maintaining a positive psychological state, like lowering stress levels, curbing modifiable behavioral practices, and achieving physical states, like working to reduce “blockages” in the heart.

*“I learned to calm down and just let it roll, you know, because too much stress or whatever cause your chest pains and everything and make you feel like [you’re] having a hard day.”*

One participant believed that heart conditions were transmissible (“catching heart disease”) and another participant confounded the effects of “spicy foods”, which can potentially cause gastro-intestinal discomfort, with heart disease.

Participants also reflected how a specific event, e.g., the COVID-19 pandemic, triggered suboptimal behavioral health habits (e.g., use of drugs, alcohol) and suboptimal food choices, like excess snacking. Participants also expressed that having disclosed their HIV status had a positive impact on their health. Participants expressed how a diagnosis of HIV can be a catalyst for awareness and knowledge about general health and about living with HIV.

*”I feel that positive people are more in tune, updated, interested in their heart health than people who are not positive.”*

*“ … positive people are more up [on things]. We really have to take care of ourselves more because we get everything. We look at everything. All parts.”*

**Table 1:** Participants’ heart health knowledge.

Question/Responses	Higher Order Themes	Example Quotes
*What “know about your heart health?”*		
Eating/avoiding harmful foods Taking vitamins Keeping positive psychological state/behaviors Physical state important (blockages, weight) Pandemic lead to suboptimal health behaviors (drugs, alcohol) Pandemic lead to suboptimal food choices Pandemic lead to good relationships HIV disclosure had positive impact on health HIV disclosure occurred early in diagnosis Offered advice to self and others HIV diagnosis lead to knowledge/awareness CVD warning signs are important to know Are you interested in learning more about heart health? Yes (all)	Preventative Measures Event Triggered Suboptimal Health Behaviors And Habits Event Triggered Good Relationships Effect of Disclosure Providing/Sharing Support to Self/Other Diagnosis Catalyst for Knowledge/Awareness	*“I learned to calm down and just let it roll, you know, because too much stress or whatever cause your chest pains and everything and make you feel like [you’re] having a hard day.”* *“I feel that positive people are more in tune, updated, interested in their heart health than people who are not positive.”* *“Exactly you know, but positive people are more up [on things]. We really have to take care of ourselves more because we get everything. We look at everything. All parts.”*

### 3.2. Willingness to Know More about Heart Health: (See Table 1)

All who responded (n = 17; 100%), were willing to learn more about how to keep their heart healthy. Specifically, participants wanted more information on how food, exercise, stress awareness, and keying into warning signs could prevent suboptimal heart health. Regarding receiving heart health information, of those who responded to the question, participants expressed willingness to read the literature (n = 3) and other materials, hearing it from the doctor directly (n = 2) or from a health educator (n = 3). Two participants were opposed to reading the literature.

### 3.3. Acceptance of Medications for Heart: (See Table 2)

When asked whether they would take medications for their heart health, all who responded (n = 16; 100%) were willing to accept prescribed medications to support their heart health: two (2) preferred to pick up their medications in person, due to wanting to consult with a professional, particularly about medications taken for their other health conditions.

*“I’d pick up myself, because … I’m not sure … I’m not sure about my [other] medications.”*

*“Well, I think that people need medical attention. And I would pick up.”*

**Table 2:** Participants’ acceptance of different interventions to improve heart health.

Question/Responses	Higher Order Themes	Examples
If medication were needed, would you accept and how?		
Yes Yes, via pharmacy (any mode) Yes, via pharmacy delivery Yes, only in-person (preferred)	Convenience an important factor Meeting with pharmacist important due to other co-morbidities	*“I’d pick up myself, because … I’m not sure … I’m not sure about my [other] medications.”* *“Well, I think that people need medical attention. And I would pick up.*
If exercise were recommended, how would you prefer to do it?		
Yes, Interested Indoor Activities Outdoor Activities Exercise at/through Work	Convenience of location an important factor Weather (heat) important factor	*“Oh, thank God, I get some exercise [at work]”* *“ … too hot [to exercise outdoors]”*
How would you prefer to receive more information about heart health?		
Preferred Mode Obtaining Heart Health Info Doctor’s Input Health Educator Reading/Literature Avoid Reading/Literature	Provided by healthcare professional/para-professional preferred Reading materials also preferred	*“ … [I] like the doctor to explain the situation”*

### 3.4. Acceptance of Exercise for Heart Health: (See Table 2)

All participants were willing to consider exercise to improve their heart health. Of those who responded (n = 11), most (64%) specified indoor activities, like attending a gym or availing themselves of opportunities to exercise at work. The outdoor heat/sun and the time-saving aspect and convenience of exercising at work were important considerations, as well.

### 3.5. Experiences of Stigma: (See Table 3)

When asked about experiences of stigma and multiple/intersectional stigma, three participants had not recalled any experiences; the majority (13; 68%) reported experiences of enacted stigma related to their HIV diagnosis and three endorsed an intersectional experience. Participants described stigma in singular words (“*isolation*”, “*judgement*”, “*ignorance*”) or elaborated on their experiences (see Table 3).

**Table 3:** Participant’s stigma experiences.

Question/Responses	Higher Order Themes	Examples
So, because of who you are, what negative experiences, stigma, or discriminations have you experienced in healthcare?		
NO YES: Intersectional Stigma (Ethnicity, Substance Use, Homelessness) YES: HIV-Related Stigma	Experience of Stigma Described Persistence of Stigma in Current Times Intersectional Stigma	*“isolation, judgement, ignorance”* *“judgement”* *“It’s just so heavy”* *“When I first learned that I was positive, people weren’t as knowledgeable as they are now. You know, although you working in the health system, they still were scared because sometimes people did, you know, have stigma in common. Yeah. Yes. There was like back back way; [now] these people got more knowledge about it and they understand that they can’t get from just touching one of us, you know, being around us and stuff like that.“* *Stigma. Yes. Is [it] is still here. It’s still here. And that’s awful because we’ve been living with this disease for so long and it shouldn’t be.* *“ … my active addiction at that time.”* *“I caught HIV in the streets—prostitution in 2016 … I, too, didn’t want to live any more. I couldn’t believe it was positive is the first positive test I ever had, and I’m glad. For my partner, he helped me get through it because I really didn’t understand it. I didn’t want to live anymore. I was homeless for like ten years. And he used to talk about me and stuff like that. I was real skinny and everything, but, you know, I was on drugs. I had a bad drug habit, you know … for a while. But finally, I got treatment.“*
	Resilience through Support	*“And [partner] used to come out and pick me up and take me to the clinic and stuff. And he helped me get into a program and get on medication and do the things and let me know that, you know, it’s going to be okay.”*
	Resilience through Self	*“ … you see how people … will hit you below the belt … would call and tell me, ‘you have HIV’. And that, to me that hits me in my gut but I don’t carry stigma with me. I’m good. That’s it. Thank you for letting me share that.”* *“ … makes me stronger …”* *“ … [I used ask others,] ‘do you know your status?’ …”*
	Resilience through Faith/Religion/Spirituality	*“God loves me. God bless them and help me. That’s all I could do. And I just kept on moving, you know”*

The HIV diagnosis, combined with enacted stigma related to their HIV diagnosis, was also expressed.

*“It’s just so heavy”.*

They also described the support from others that helped them:

And *“[partner] used to come out and pick me up and take me to the clinic and stuff. And he helped me get into a program and get on medication and do the things and let me know that, you know, it’s going to be okay.”*

Faith, spirituality/religion was also a source of support in helping women move past these experiences.

*“God loves me. God bless them and help me. That’s all I could do. And I just kept on moving, you know.”*

Participants expressed how, despite the progress that had been made regarding people’s understanding of HIV and how HIV is and is not transmitted, even in current times, stigmatizing experiences persisted.

*“When I first learned that I was positive, people weren’t as knowledgeable as they are now … they understand [now] that they can’t get [it] from just touching one of us, you know, being around us and stuff like that … “*

*“Stigma … It’s still here. And that’s awful because we’ve been living with this disease for so long and it shouldn’t be.*

Participants also described the journey from first being diagnosed, building up resilience to their stigmatizing experiences, then moving beyond the negative experiences:

*“ … you see how people … will hit you below the belt …[people] would call and tell me, ‘you have HIV’. And that, to me that hits me in my gut but I don’t carry stigma with me. I’m good. That’s it. Thank you for letting me share that.”*

*“[the experience] makes me stronger …”*

Participants were then asked to share negative and discriminatory experiences related to their other multiple, marginalized identities, or intersectional stigmas.

*“… didn’t want to live any more. I couldn’t believe it [the HIV test] was positive … [it] is the first positive test I ever had, and I’m glad. For my partner, he helped me get through it because I really didn’t understand it. I didn’t want to live anymore. I was homeless for like ten years. And he used to talk about me and stuff like that. I was real skinny and everything, but, you know, I was on drugs. I had a bad drug habit, you know, around for a while. But finally, I got treatment.*

## 4. Discussion

People with HIV are living longer, and as such, are developing more co-morbidities like cardiovascular disease [10,14]. WWH, specifically, are at an even higher risk for cardiac disease, compared to their male counterparts [16]. Being informed about health knowledge is important as a CVD prevention intervention. Indeed, heart health knowledge has been tied to low interest in reducing CVD risk [8].

### 4.1. Heart Health Knowledge and Willingness to Learn More

The present study found that WWH were able to articulate knowledge about heart health and CVD risk; however, as reported in a prior study, knowledge may not be sufficient [22]. A previously reported study found that PWH were knowledgeable about CVD risk, they did not, however, perceive they were at risk for cardiac conditions [22]. Overall, participants articulated that prevention is important. They also articulated that certain events (like the COVID-19 pandemic), triggered suboptimal health behaviors and habits for their heart health and that their HIV diagnosis was a catalyst for understanding more about their overall health (“*I learned to calm down and just let it roll, you know, because too much stress or whatever cause your chest pains and everything and make you feel like I’m having a hard day.*”).

As summarized from interviews of the present study, WWH were willing to learn more about heart health and take additional measures to reduce their CVD risk, like making dietary changes and exercising. At the time of this writing, limited programs were available at their local HIV clinic. As the population of people with HIV is aging, clinics and health systems have begun to incorporate onsite programs such as the US-based *Golden Compass* program. The program, which is implemented in a safety net clinic on the US Western Coast, is geared for older adults (≥50) with HIV. Two of the *Golden Compass* program modules, “Heart and Mind”, which includes cardiology and cognitive evaluations, and brain health classes; and “Bones and Strength”, which focuses on bone health and on-site geriatric consultation [23], could address a gap in available programs that serve aging PWH.

### 4.2. Medications for Heart Health

As people with HIV are more likely to develop co-morbidities with age, like diabetes, hypertension and cardiovascular disease [10,14], they are also taking more medications to manage these co-morbid conditions [24]. The present study found that WWH would consider taking additional prescribed medications to improve their cardiac health; there was some concern, however, about having medications delivered, as opposed to retrieving them in person, where a pharmacist could be consulted. Overall, both convenience and the ability to receive expert advice (like from a pharmacist) when medications are picked up was very important for them. One intervention implemented in Europe (Spain), linked patients with HIV to a pharmacy-based program and successfully improved their blood lipid levels and hypertension [25]

### 4.3. Exercise for Heart Health

In the present study, participants were willing to exercise specifically to improve their heart health. The majority preferred the convenience of indoor exercise, like a gym to avoid the heat, or for an option at their workplace, which was more convenient. As previously mentioned, the US-based Golden Compass program [23] is a model that could be implemented in similar safety net clinics. Similar to how participants responded to taking medications for their heart health, most participants opted for convenience (like having access to exercise at work); however, other considerations (e.g., indoor exercise to avoid the heat) were important as well.

### 4.4. Method of Receiving Information to Improve Heart Health

When asked how they would prefer to receive information about their heart health, participants overall wanted to receive information from professionals and para-professionals.

### 4.5. Experiences of Stigma

We probed participants about their experiences with HIV-related stigma and other stigma. The majority (16; 84%) expressed experiencing some kind of stigma. In general, participants described clearly their stigma experience, some of which was intersectional in nature (e.g., racial/ethnic minority status, substance use, homelessness), and that although improvements in HIV knowledge and awareness had been made, societally, stigma still persisted. Participants also expressed that they developed resilience through support, faith/religion, and spirituality. Many participants experienced stigma earlier on in their diagnosis, and explained that even though, in general, people understood more about HIV in society and that HIV was not passed through casual contact, the experience of stigma persisted. This parallels what was reported in another qualitative study by Lekas et al. (2006) [26].

Participants were then asked specifically about their intersectional stigma experiences. One participant described contracting HIV while engaged in sex work and being judged because she developed a “bad drug habit” and was “skinny” and “homeless”. We found faith/spirituality/religion was also a source of support that helped women cope with their experiences of stigma. Results from a meta-analysis demonstrated that spirituality/religion was an adaptation mechanism for women with HIV [27].

To our knowledge, this is one of the first studies to explore the role of stigma within the context of cardiovascular risk and disease.

## 5. Limitations

This was a small study, conducted during the COVID-19 pandemic and the study encountered several logistical and recruitment hurdles. The findings provide rich insights into the challenges faced by WWH and will inform investigators conducting similar research about the areas that need attention—helping to bridge the knowledge gap regarding CVD risk prevention for this population.

## 6. Conclusions

Among this cohort of racial and ethnic minoritized women with HIV living in Southern Florida, women shared their HIV and other related stigmatizing experiences that persisted for several years, and the resilience they developed. Our findings suggest the resilience these women acquired may have mitigated the harmful effects of the intersecting stigmas they experienced. Overall, we found that women had some basic knowledge about improving heart health; however, they wanted to understand more about prevention to reduce their CVD risk and were willing to take additional measures, especially if recommended or prescribed by a healthcare provider. Women also expressed that convenience was an important factor for them (like delivery of medications); however, at times, an in-person encounter with a pharmacist was also important to them, to ensure they understood considerations with taking other medications. We conclude that clinics that serve aging women with HIV should include more education and programming about heart health that is customizable to their needs. Additional work is needed, however, to further understand the relationship between CVD risk, stigma, resilience, and other important factors, amongst racial and ethnic minoritized women with HIV.

## Data Availability

The datasets presented in this article are not readily available due to restrictions, as stated the Department of Health and Human Services. Requests to access the datasets should be directed to http://privacy.med.miami.edu/employees/data-use-agreements.
